# 3,6,8-Trihy­droxy-3,4,5,7-tetra­methyl-3,4-dihydro­isocoumarin

**DOI:** 10.1107/S1600536811021751

**Published:** 2011-06-18

**Authors:** Yi-Wen Tao, Yun Wang

**Affiliations:** a School of Basic Science, Guangzhou Medical College, Guangzhou 510182, People’s Republic of China; b Guangdong Institute for Drug Control, Guangzhou 510180, People’s Republic of China

## Abstract

In the title compound, C_13_H_16_O_5_, one of the three hy­droxy groups is involved in intra­molecular O—H⋯O hydrogen bonds. The other two hy­droxy groups contribute to the three-dimensional hydrogen-bonding network, which consolidates the crystal packing.

## Related literature

For related structures, see: Wang *et al.* (2003 ▶); Krohn *et al.* (1997 ▶).
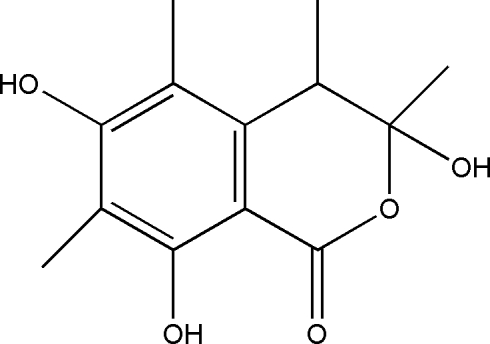


## Experimental

### 

#### Crystal data

C_13_H_16_O_5_
*M* *_r_* = 252.26
Orthorhombic, 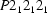

*a* = 7.4731 (3) Å
*b* = 9.9742 (7) Å
*c* = 16.4066 (12) Å
*V* = 1222.92 (13) Å^3^
*Z* = 4
Cu *K*α radiation
μ = 0.88 mm^−1^
*T* = 150 K
0.40 × 0.20 × 0.20 mm

#### Data collection

Oxford Diffraction Xcalibur diffractometer with Onyx (Nova) detector
Absorption correction: multi-scan (*CrysAlis PRO*; Oxford Diffraction, 2006 ▶) *T*_min_ = 0.719, *T*_max_ = 0.843
11941 measured reflections
2199 independent reflections
2165 reflections with *I* > 2σ(*I*)
*R*_int_ = 0.029

#### Refinement

*R*[*F* ^2^ > 2σ(*F* ^2^)] = 0.025
*wR*(*F* ^2^) = 0.069
*S* = 1.07
2199 reflections
171 parameters
H-atom parameters constrained
Δρ_max_ = 0.22 e Å^−3^
Δρ_min_ = −0.14 e Å^−3^
Absolute structure: Flack (1983 ▶), 883 Friedel pairs
Flack parameter: 0.13 (15)

### 

Data collection: *CrysAlis PRO* (Oxford Diffraction, 2006 ▶); cell refinement: *CrysAlis PRO*; data reduction: *CrysAlis PRO*; program(s) used to solve structure: *SHELXTL* (Sheldrick, 2008 ▶); program(s) used to refine structure: *SHELXTL*; molecular graphics: *OLEX2* (Dolomanov *et al.*, 2009 ▶); software used to prepare material for publication: *OLEX2*.

## Supplementary Material

Crystal structure: contains datablock(s) I, global. DOI: 10.1107/S1600536811021751/cv5104sup1.cif

Structure factors: contains datablock(s) I. DOI: 10.1107/S1600536811021751/cv5104Isup2.hkl

Supplementary material file. DOI: 10.1107/S1600536811021751/cv5104Isup3.cml

Additional supplementary materials:  crystallographic information; 3D view; checkCIF report

## Figures and Tables

**Table 1:** Hydrogen-bond geometry (Å, °)

*D*—H⋯*A*	*D*—H	H⋯*A*	*D*⋯*A*	*D*—H⋯*A*
O4—H4⋯O5	0.84	1.87	2.6040 (13)	145
O2—H2⋯O5^i^	0.84	1.95	2.7851 (13)	172
O3—H3⋯O2^ii^	0.84	2.15	2.8942 (14)	148

Symmetry codes: (i) 
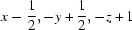
; (ii) 
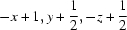
.

**Fig. 1.**
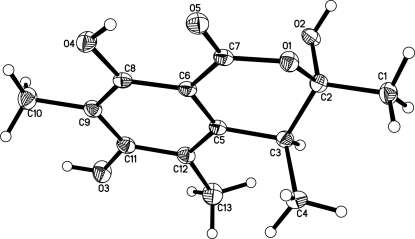
The molecular structure of (I) with the atom-labelling scheme. Displacement ellipsoids are drawn at the 50% probability level.

## supplementary crystallographic information

### Comment

The title compound, C_13_H_16_O_5_ (I), also known as sclerotinin A, was isolated from culture filtrate of the endophytic fungus phomposis *sp* from mangrove trees from the coast of the South China Sea. Herewith we present the structure of sclerotinin A.

The crystal structure of the title compound is shown in Fig. 1. The titel compound crystalizes in orthorhombic cell setting P2(1)2(1)2(1) space group, containing four formula units in the unit cell. As can be found,

In (I), all non-H atoms, except C1, C2, C4 and O2, are coplanar with the mean deviation of 0.039 (2) Å. The C2/C3/C5/C6/C7/O1 heterocycle exhibits an envelope configuration with C2 out of the plane formed by the other five atoms 0.308 (2) Å. All the bond lengths and angles are comparable with those found in similar compounds reported previously (Wang *et al.*, 2003; Krohn *et al.*, 1997).

In the crystal structure, intermolecular O—H···O hydrogen bonds (Table 1) generate three-dimensional network, which consolidate the crystal packing.

### Experimental

A strain of the fungus phomposis *sp* was isolated from the mangrove tree, Zhanjiang, and was stored at the Department of Applied Chemistry, Zhongshan University, Guangzhou, China. Starter cultures (from Professor Shining Zhou) were maintained on cornmeal seawater agar. Plugs of agar supporting mycelial growth were cut and transferred aseptically to a 500 ml Erlenmeyer flask containing 300 ml of liquid medium (glucose 1%, peptone 0.2%, yeast extract 0.1%, NaCl 0.25%, pH 7.0). The flask was incubated at 303 K on a rotary shaker for 3 days. The mycelium was aseptically incubated at 303 K for 5 weeks. The cultures (130 *L*) were filtered through cheesecloth. The filtrate was concentrated to 3 *L* below 328 K and extracted several times by shaking with twofold volume of the ethyl acetate. The combined extracts were concentrated and chromatographed on silica gel using a gradient elution from petroleum to ethyl acetate, obtaining compound sclerotinin A from the 30% ethyl acetate/petroleum ether fraction. Crystals of the title compound suitable for single-crystal X-ray diffraction analysis were obtained by recrystallization from methanol.

### Refinement

All the H atoms were geometrically positioned (C—H 0.98 A°, O—H 0.84 A°),  and were refined as riding, with *U*_iso_(H) = 1.2-1.5 *U*_eq_(C, O).

### Crystal data

**Table:**

C_13_H_16_O_5_	*F*(000) = 536
*M**_r_* = 252.26	*D*_x_ = 1.370 Mg m^−^^3^
Orthorhombic, *P*2_1_2_1_2_1_	Cu *K*α radiation, λ = 1.54178 Å
Hall symbol: P 2ac 2ab	Cell parameters from 1678 reflections
*a* = 7.4731 (3) Å	θ = 5.2–68.2°
*b* = 9.9742 (7) Å	µ = 0.88 mm^−^^1^
*c* = 16.4066 (12) Å	*T* = 150 K
*V* = 1222.92 (13) Å^3^	Block, colourless
*Z* = 4	0.40 × 0.20 × 0.20 mm

### Data collection

**Table:**

Oxford Diffraction Xcalibur diffractometer with Onyx (Nova) detector	2199 independent reflections
Radiation source: Nova (Cu) X-ray Source	2165 reflections with *I* > 2σ(*I*)
mirror	*R*_int_ = 0.029
Detector resolution: 8.2417 pixels mm^-1^	θ_max_ = 68.2°, θ_min_ = 5.2°
ω scans	*h* = −7→8
Absorption correction: multi-scan (*CrysAlis PRO*; Oxford Diffraction, 2006)	*k* = −11→11
*T*_min_ = 0.719, *T*_max_ = 0.843	*l* = −19→18
11941 measured reflections	

### Refinement

**Table:**

Refinement on *F*^2^	Hydrogen site location: inferred from neighbouring sites
Least-squares matrix: full	H-atom parameters constrained
*R*[*F*^2^ > 2σ(*F*^2^)] = 0.025	*w* = 1/[σ^2^(*F*_o_^2^) + (0.0427*P*)^2^ + 0.1914*P*] where *P* = (*F*_o_^2^ + 2*F*_c_^2^)/3
*wR*(*F*^2^) = 0.069	(Δ/σ)_max_ < 0.001
*S* = 1.07	Δρ_max_ = 0.22 e Å^−^^3^
2199 reflections	Δρ_min_ = −0.14 e Å^−^^3^
171 parameters	Extinction correction: *SHELXL*, Fc^*^=kFc[1+0.001xFc^2^λ^3^/sin(2θ)]^-1/4^
0 restraints	Extinction coefficient: 0.0039 (7)
Primary atom site location: structure-invariant direct methods	Absolute structure: Flack (1983), 883 Friedel pairs
Secondary atom site location: difference Fourier map	Flack parameter: 0.13 (15)

### Special details

**Table:**

Geometry. All e.s.d.'s (except the e.s.d. in the dihedral angle between two l.s. planes) are estimated using the full covariance matrix. The cell e.s.d.'s are taken into account individually in the estimation of e.s.d.'s in distances, angles and torsion angles; correlations between e.s.d.'s in cell parameters are only used when they are defined by crystal symmetry. An approximate (isotropic) treatment of cell e.s.d.'s is used for estimating e.s.d.'s involving l.s. planes.
Refinement. Refinement of *F*^2^ against ALL reflections. The weighted *R*-factor *wR* and goodness of fit *S* are based on *F*^2^, conventional *R*-factors *R* are based on *F*, with *F* set to zero for negative *F*^2^. The threshold expression of *F*^2^ > σ(*F*^2^) is used only for calculating *R*-factors(gt) *etc*. and is not relevant to the choice of reflections for refinement. *R*-factors based on *F*^2^ are statistically about twice as large as those based on *F*, and *R*- factors based on ALL data will be even larger.

### Fractional atomic coordinates and isotropic or equivalent isotropic displacement parameters (Å^2^)

**Table:**

	*x*	*y*	*z*	*U*_iso_*/*U*_eq_	
O1	0.50038 (12)	0.41865 (9)	0.52036 (5)	0.0280 (2)	
O2	0.26458 (13)	0.35748 (9)	0.43613 (5)	0.0295 (2)	
H2	0.2619	0.2802	0.4564	0.044*	
O5	0.77901 (13)	0.38899 (10)	0.48436 (6)	0.0319 (2)	
C8	0.74508 (17)	0.54758 (12)	0.34132 (7)	0.0242 (3)	
O3	0.53133 (15)	0.79291 (11)	0.20856 (6)	0.0397 (3)	
H3	0.6232	0.7996	0.1793	0.060*	
O4	0.90385 (13)	0.48275 (9)	0.34758 (6)	0.0319 (2)	
H4	0.9037	0.4346	0.3896	0.048*	
C9	0.72242 (18)	0.63290 (12)	0.27480 (7)	0.0273 (3)	
C6	0.60748 (17)	0.53081 (12)	0.39893 (7)	0.0228 (3)	
C12	0.42314 (18)	0.69223 (13)	0.32709 (8)	0.0280 (3)	
C7	0.63436 (17)	0.44348 (12)	0.46828 (7)	0.0244 (3)	
C11	0.56276 (19)	0.70428 (13)	0.26991 (8)	0.0284 (3)	
C5	0.44607 (16)	0.60298 (12)	0.39092 (7)	0.0231 (3)	
C3	0.30879 (17)	0.58956 (12)	0.45805 (7)	0.0253 (3)	
H3A	0.1876	0.6020	0.4333	0.030*	
C4	0.3370 (2)	0.70133 (14)	0.52100 (9)	0.0355 (3)	
H4C	0.4485	0.6850	0.5509	0.053*	
H4B	0.2364	0.7024	0.5593	0.053*	
H4A	0.3443	0.7880	0.4930	0.053*	
C10	0.8647 (2)	0.64783 (16)	0.21033 (8)	0.0379 (3)	
H10A	0.8158	0.6215	0.1573	0.057*	
H10B	0.9668	0.5903	0.2238	0.057*	
H10C	0.9041	0.7415	0.2080	0.057*	
C2	0.31545 (17)	0.44997 (13)	0.49601 (7)	0.0257 (3)	
C1	0.20825 (19)	0.43331 (14)	0.57328 (8)	0.0333 (3)	
H1C	0.2544	0.4943	0.6151	0.050*	
H1B	0.2184	0.3406	0.5925	0.050*	
H1A	0.0823	0.4542	0.5624	0.050*	
C13	0.2588 (2)	0.77862 (17)	0.31733 (10)	0.0434 (4)	
H13B	0.1546	0.7315	0.3396	0.065*	
H13A	0.2390	0.7972	0.2594	0.065*	
H13C	0.2759	0.8633	0.3466	0.065*	

### Atomic displacement parameters (Å^2^)

**Table:**

	*U*^11^	*U*^22^	*U*^33^	*U*^12^	*U*^13^	*U*^23^
O1	0.0301 (5)	0.0298 (5)	0.0240 (4)	0.0021 (4)	−0.0010 (4)	0.0057 (3)
O2	0.0414 (5)	0.0225 (4)	0.0245 (4)	−0.0063 (4)	−0.0003 (4)	0.0013 (3)
O5	0.0311 (5)	0.0304 (5)	0.0341 (5)	0.0052 (4)	−0.0028 (4)	0.0079 (4)
C8	0.0268 (6)	0.0197 (5)	0.0262 (6)	0.0001 (5)	−0.0028 (5)	−0.0033 (5)
O3	0.0429 (6)	0.0429 (5)	0.0332 (5)	0.0073 (5)	0.0039 (4)	0.0184 (4)
O4	0.0303 (5)	0.0333 (5)	0.0322 (5)	0.0061 (4)	0.0016 (4)	0.0053 (4)
C9	0.0333 (7)	0.0245 (6)	0.0240 (6)	−0.0027 (5)	0.0005 (5)	−0.0006 (5)
C6	0.0287 (6)	0.0180 (5)	0.0218 (6)	−0.0013 (5)	−0.0018 (4)	−0.0017 (4)
C12	0.0319 (7)	0.0245 (6)	0.0278 (6)	0.0014 (5)	−0.0017 (5)	0.0031 (5)
C7	0.0299 (6)	0.0195 (5)	0.0239 (6)	−0.0007 (5)	−0.0021 (5)	−0.0017 (5)
C11	0.0364 (7)	0.0235 (6)	0.0253 (6)	−0.0007 (5)	−0.0022 (5)	0.0042 (5)
C5	0.0259 (6)	0.0193 (5)	0.0240 (5)	−0.0013 (5)	−0.0020 (5)	−0.0015 (5)
C3	0.0271 (6)	0.0229 (6)	0.0259 (6)	0.0018 (5)	−0.0001 (5)	0.0017 (5)
C4	0.0423 (7)	0.0277 (6)	0.0364 (7)	0.0009 (6)	0.0059 (6)	−0.0067 (5)
C10	0.0398 (8)	0.0414 (7)	0.0326 (7)	0.0026 (6)	0.0068 (6)	0.0077 (6)
C2	0.0281 (6)	0.0263 (6)	0.0226 (6)	−0.0004 (5)	−0.0016 (5)	−0.0002 (5)
C1	0.0375 (7)	0.0366 (7)	0.0259 (6)	−0.0019 (6)	0.0041 (5)	0.0035 (5)
C13	0.0406 (8)	0.0431 (8)	0.0464 (8)	0.0119 (7)	0.0008 (7)	0.0166 (6)

### Geometric parameters (Å, °)

**Table:**

O1—C7	1.3395 (15)	C5—C3	1.5111 (17)
O1—C2	1.4721 (16)	C3—C2	1.5260 (17)
O2—C2	1.4003 (15)	C3—C4	1.5343 (18)
O2—H2	0.8400	C3—H3A	1.0000
O5—C7	1.2384 (16)	C4—H4C	0.9800
C8—O4	1.3552 (16)	C4—H4B	0.9800
C8—C9	1.3942 (17)	C4—H4A	0.9800
C8—C6	1.4067 (18)	C10—H10A	0.9800
O3—C11	1.3601 (15)	C10—H10B	0.9800
O3—H3	0.8400	C10—H10C	0.9800
O4—H4	0.8400	C2—C1	1.5088 (17)
C9—C11	1.3917 (19)	C1—H1C	0.9800
C9—C10	1.5073 (18)	C1—H1B	0.9800
C6—C5	1.4108 (18)	C1—H1A	0.9800
C6—C7	1.4469 (16)	C13—H13B	0.9800
C12—C5	1.3851 (17)	C13—H13A	0.9800
C12—C11	1.4082 (19)	C13—H13C	0.9800
C12—C13	1.509 (2)		
			
C7—O1—C2	119.30 (9)	C3—C4—H4C	109.5
C2—O2—H2	109.5	C3—C4—H4B	109.5
O4—C8—C9	117.19 (11)	H4C—C4—H4B	109.5
O4—C8—C6	122.16 (11)	C3—C4—H4A	109.5
C9—C8—C6	120.65 (11)	H4C—C4—H4A	109.5
C11—O3—H3	109.5	H4B—C4—H4A	109.5
C8—O4—H4	109.5	C9—C10—H10A	109.5
C11—C9—C8	117.48 (12)	C9—C10—H10B	109.5
C11—C9—C10	120.93 (11)	H10A—C10—H10B	109.5
C8—C9—C10	121.59 (12)	C9—C10—H10C	109.5
C8—C6—C5	120.11 (11)	H10A—C10—H10C	109.5
C8—C6—C7	119.90 (11)	H10B—C10—H10C	109.5
C5—C6—C7	119.94 (11)	O2—C2—O1	107.79 (10)
C5—C12—C11	117.83 (12)	O2—C2—C1	111.88 (10)
C5—C12—C13	123.22 (12)	O1—C2—C1	104.30 (10)
C11—C12—C13	118.93 (12)	O2—C2—C3	107.81 (9)
O5—C7—O1	115.81 (10)	O1—C2—C3	109.57 (10)
O5—C7—C6	123.57 (11)	C1—C2—C3	115.22 (11)
O1—C7—C6	120.61 (11)	C2—C1—H1C	109.5
O3—C11—C9	121.54 (12)	C2—C1—H1B	109.5
O3—C11—C12	114.87 (12)	H1C—C1—H1B	109.5
C9—C11—C12	123.58 (11)	C2—C1—H1A	109.5
C12—C5—C6	120.27 (11)	H1C—C1—H1A	109.5
C12—C5—C3	121.60 (11)	H1B—C1—H1A	109.5
C6—C5—C3	117.86 (10)	C12—C13—H13B	109.5
C5—C3—C2	110.86 (10)	C12—C13—H13A	109.5
C5—C3—C4	109.44 (10)	H13B—C13—H13A	109.5
C2—C3—C4	112.57 (11)	C12—C13—H13C	109.5
C5—C3—H3A	107.9	H13B—C13—H13C	109.5
C2—C3—H3A	107.9	H13A—C13—H13C	109.5
C4—C3—H3A	107.9		
			
O4—C8—C9—C11	−177.25 (11)	C11—C12—C5—C6	2.13 (18)
C6—C8—C9—C11	2.70 (17)	C13—C12—C5—C6	−176.37 (13)
O4—C8—C9—C10	2.78 (17)	C11—C12—C5—C3	176.12 (11)
C6—C8—C9—C10	−177.27 (11)	C13—C12—C5—C3	−2.4 (2)
O4—C8—C6—C5	178.45 (10)	C8—C6—C5—C12	−1.01 (17)
C9—C8—C6—C5	−1.50 (17)	C7—C6—C5—C12	176.51 (11)
O4—C8—C6—C7	0.92 (17)	C8—C6—C5—C3	−175.22 (10)
C9—C8—C6—C7	−179.03 (11)	C7—C6—C5—C3	2.31 (16)
C2—O1—C7—O5	−164.73 (11)	C12—C5—C3—C2	153.52 (11)
C2—O1—C7—C6	16.22 (16)	C6—C5—C3—C2	−32.36 (14)
C8—C6—C7—O5	6.00 (18)	C12—C5—C3—C4	−81.73 (14)
C5—C6—C7—O5	−171.53 (11)	C6—C5—C3—C4	92.39 (13)
C8—C6—C7—O1	−175.02 (10)	C7—O1—C2—O2	70.86 (13)
C5—C6—C7—O1	7.45 (17)	C7—O1—C2—C1	−170.08 (10)
C8—C9—C11—O3	177.94 (11)	C7—O1—C2—C3	−46.21 (14)
C10—C9—C11—O3	−2.09 (19)	C5—C3—C2—O2	−64.88 (12)
C8—C9—C11—C12	−1.55 (19)	C4—C3—C2—O2	172.15 (11)
C10—C9—C11—C12	178.42 (12)	C5—C3—C2—O1	52.17 (12)
C5—C12—C11—O3	179.62 (11)	C4—C3—C2—O1	−70.79 (13)
C13—C12—C11—O3	−1.81 (19)	C5—C3—C2—C1	169.38 (11)
C5—C12—C11—C9	−0.9 (2)	C4—C3—C2—C1	46.42 (15)
C13—C12—C11—C9	177.71 (13)		

### Hydrogen-bond geometry (Å, °)

**Table:**

*D*—H···*A*	*D*—H	H···*A*	*D*···*A*	*D*—H···*A*
O4—H4···O5	0.84	1.87	2.6040 (13)	145
O2—H2···O5^i^	0.84	1.95	2.7851 (13)	172
O3—H3···O2^ii^	0.84	2.15	2.8942 (14)	148

Symmetry codes: (i) *x*−1/2, −*y*+1/2, −*z*+1; (ii) −*x*+1, *y*+1/2, −*z*+1/2.
